# Liposomal Drug Delivery against *Helicobacter pylori* Using Furazolidone and N-Acetyl Cysteine in Augmented Therapy

**DOI:** 10.3390/pharmaceutics16091123

**Published:** 2024-08-26

**Authors:** Muhammad Irfan Alam, Timothy Paget, Najla Yussuf Moosa, Husein Alghurairy, Amal Ali Elkordy

**Affiliations:** 1 School of Pharmacy and Pharmaceutical Sciences, Faculty of Health Sciences and Wellbeing, University of Sunderland, Sunderland SR1 3SD, UK; drfomialam@gmail.com; 2 School of Medicine, Faculty of Health Sciences and Wellbeing, University of Sunderland, Sunderland SR1 3SD, UK; timothy.paget@sunderland.ac.uk (T.P.); najla.moosa@sunderland.ac.uk (N.Y.M.); 3 Dudley Group NHS Foundation Trust, Dudley DY1 2HQ, UK; husein.alghurairy@nhs.net

**Keywords:** mucus penetration, liposomes, N-acetyl cysteine, *H. pylori*, furazolidone, augmented therapy

## Abstract

*Helicobacter pylori* (*H. pylori*) infection is a significant global health concern, affecting approximately 50% of the world’s population and leading to gastric ulcers, gastritis, and gastric cancer. The increase in antibiotic resistance has compromised the efficacy of existing therapeutic regimens, necessitating novel approaches for effective eradication. This study aimed to develop a targeted liposomal drug delivery system incorporating furazolidone and N-acetylcysteine (NAC) to enhance mucopenetration and improve *Helicobacter pylori* eradication. Liposomes were formulated with furazolidone, NAC, and Pluronic F-127 using a modified reverse-phase evaporation technique. The formulations were categorized based on charge as neutral, negative, and positive and tested for mucopenetration using a modified silicon tube method with coumarin-6 as a fluorescent marker. The encapsulation efficiency and particle size were analyzed using HPLC and an Izon q-nano particle size analyzer. The results indicated that charged liposomes showed a higher encapsulation efficiency than neutral liposomes with Pluronic F-127. Notably, combining furazolidone with 1% NAC achieved complete eradication of *H. pylori* in 2.5 h, compared to six hours without NAC. The findings of this study suggest that incorporating NAC and Pluronic F-127 into liposomal formulations significantly enhances mucopenetration and antimicrobial efficacy.

## 1. Background

*Helicobacter pylori* (*H. pylori*) is a Gram-negative bacterium that colonizes the human stomach, causing chronic gastritis and peptic ulcers, and is strongly associated with the development of gastric cancer [1,2,3]). The pathogenicity of the bacterium is primarily attributed to its ability to survive in the acidic environment of the stomach, facilitated by the enzyme urease, which hydrolyzes urea to produce ammonia, thereby neutralizing stomach acid. Additionally, *H. pylori* adheres to the gastric epithelium, avoiding an immune response and creating a chronic inflammatory state that can lead to tissue damage [4,5]. Approximately 50% of the world’s population is infected with *H. pylori* [3,6]. It is most common in developing countries, with up to 70% of the population carrying some strains of *H. pylori* [7]. In the U.K., approximately 40% of people are infected with *H. pylori* [8].

The treatment of *H. pylori* infections has become increasingly challenging because of the rise in antibiotic resistance. Standard triple therapy, which typically includes a proton pump inhibitor and two antibiotics, often fails because of resistance to commonly used antibiotics, such as clarithromycin and metronidazole [6,9,10]. The principal drugs investigated in this study were furazolidone and N-acetylcysteine (NAC). Furazolidone is a yellow, crystalline, odorless drug with a molecular weight of 225.158 g/mol and limited water solubility of 4 mg/100 mL [11]. However, it is readily soluble in acetonitrile, making it an effective broad-spectrum antibiotic that covers most Gram-negative and Gram-positive microorganisms by causing DNA damage through crosslinking [12,13]. Furazolidone intercalates into the bacterial DNA, leading to the formation of crosslinks that prevent replication and transcription, ultimately causing cell death. It is well absorbed orally, distributed into tissues, including the gastrointestinal tract, metabolized in the liver, and excreted mainly through urine and feces. Side effects include gastrointestinal upset, with rare occurrences of hypersensitivity reactions and peripheral neuropathy [14,15].

N-acetylcysteine (NAC), a derivative of cysteine with a molecular weight of approximately 163.194 g/mol, is water-soluble (1 g/5 mL), partially soluble in chloroform and ether, and is widely used as a mucolytic agent in chronic obstructive pulmonary disease (COPD). NAC reduces mucus viscosity by cleaving disulfide bonds, a process enhanced by increased pH [16,17,18,19]. NAC also destabilizes biofilms, synergizes with antibiotics, and exhibits bactericidal effects [20,21]. Its antioxidant properties help to reduce oxidative stress, which can be beneficial in managing *H. pylori* infections [17,22]. Additionally, NAC’s mucolytic effects of NAC on gastric mucus may facilitate better drug penetration and bacterial eradication [23,24,25,26,27].

Pluronic F-127 is a nonionic triblock copolymer composed of a central hydrophobic block of polypropylene oxide (PPO) flanked by two hydrophilic blocks of polyethylene oxide (PEO) [28,29]. It has very low toxicity, is colorless and odorless, has free-flowing granules, and is waxy in appearance. Its molecular weight is approximately 12,500 Daltons [30,31]. Pluronic F-127 is known for its thermoreversible gel properties, which make it suitable for various drug delivery systems [28,32]. It is compatible with almost all routes of administration, including oral, nasal, vaginal, parenteral, and topical applications [33,34].

In liposomal formulations, Pluronic F-127 plays a critical role in stabilizing liposomes by reducing surface tension and preventing aggregation, thereby ensuring the integrity of the formulation in the acidic gastric environment [28]. The hydrophilic PEO chains provided a steric barrier around the liposomes, enhancing their stability. Pluronic F-127 also improves the encapsulation efficiency of water-soluble drugs, such as furazolidone and NAC, facilitating more effective delivery to the infection site. Furthermore, it enhanced the ability of liposomes to penetrate the mucus layer of the stomach lining and adhere to the gastric epithelium where *H. pylori* resides [35]. The combination of Pluronic F-127 and NAC synergistically improves mucopenetration, and the mucolytic effect complements Pluronic F-127’s mucus navigation properties.

Liposomes are among the most widely used nanocarriers in drug delivery systems [31]. They are composed of a hydrophobic bilayer surrounding a hydrophilic core, making them suitable for various drug delivery applications [34,36,37]. Numerous liposomal formulations have already been clinically approved for treating conditions, such as fungal and viral infections and cancers, with many advances in clinical trials [36,38,39,40].

Liposomes can be classified into two main types: cationic and anionic. Cationic liposomes, which have a positive surface charge, enhance interactions with the negatively charged gastric mucosal surfaces. This increased interaction improves adhesion and facilitates effective drug delivery [36,39,41]. However, studies have indicated that cationic liposomes often exhibit higher cytotoxicity and instability. Additionally, they can be inactivated by serum components, which poses challenges for oral drug delivery [42,43].

In contrast, anionic liposomes, which carry a negative charge, demonstrate greater stability in solution and reduced aggregation compared to their cationic counterparts [36,40]. Their low cytotoxicity makes them a safer choice for extended use. Thus, the decision to use cationic or anionic liposomes involves balancing the need for effective drug delivery with the goal of minimizing potential side effects [36,41].

Mucus presents a significant barrier to drug delivery systems owing to its viscous and sticky nature. Liposomes can become trapped and immobilized within the mucus mesh network regardless of their size. The dynamic turnover of mucus, involving constant secretion and shedding, further complicates liposome transport by continuously eliminating the entrapped particles [43,44].

Several strategies have been developed to overcome the mucus barrier and enhance the diffusion and penetration of liposomes [43,45,46]. These strategies include optimizing the surface charge of liposomes to balance adhesion and penetration, as well as incorporating agents that improve mucosal permeation. By refining these approaches, it is possible to enhance the therapeutic effects of liposomal formulations and achieve a more effective mucosal drug delivery [41,44,47].

The use of furazolidone, NAC, and Pluronic F-127 in targeted delivery systems aims to improve eradication and mucopenetration [11,15]. The use of liposomes for oral drug delivery has been extensively studied. Liposomal delivery systems offer several advantages, including protection of drugs from degradation in the acidic gastric environment, prolonged release, and targeted delivery to the infection site, thereby enhancing therapeutic efficacy. The major challenge in using oral drug delivery-based liposomal vesicles for the treatment of *H*. *pylori* infection is the gastric mucosal barrier [37,48].

This study aimed to develop a liposomal formulation combining furazolidone, NAC, and Pluronic F-127 to optimize drug delivery and improve treatment outcomes in *H. pylori* infections. The expected outcomes include improved mucopenetration via NAC, which disrupts disulfide bonds within the mucus, enhances antimicrobial activity, and reduces the treatment duration. Pluronic F-127 enhances diffusion through mucus. If successful, this novel approach could significantly improve the clinical management of *H. pylori* infections, particularly in cases of antibiotic resistance.

## 2. Materials and Methods

### 2.1. Materials

Furazolidone, N-acetylcysteine (NAC), and cholesterol were purchased from Merck Life Science UK Limited, Dorset, UK. Coumarin-6, reconstituted mucin, didodecyldimethylammonium bromide 98% (DDAB), and dihexadecyl phosphate (DCP) were purchased from Fisher Scientific, Loughborough, UK. *H. pylori* strain NCTC 12455 was purchased from Culture Collection UK Health Security Agency, Salisbury, UK. 1,2-distearoyl-sn-glycero-3-phosphocholine (DSPC) was purchased from Avanti Polar Lipids, Alabaster, AL, USA.

### 2.2. Preparation of Mucopenetrative Liposomes

**Scheme 1:**
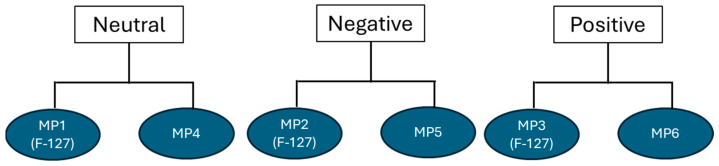
The coding for mucopenetrative liposomal formulation compositions (MP1-MP6).

Charged and neutral liposomal formulations were prepared using a modified reverse-phase evaporation (REV) technique as described below. For cationic liposomal preparation, didodecyldimethylammonium bromide 98% (DDAB) was used in the lipid mixture, whereas dihexadecyl phosphate (DCP) was used for negatively charged liposomes. However, only 1, 2-distearoyl-sn-glycero-3-phosphocholine (DSPC) and cholesterol have been used as neutral liposomes. The coding for formulations (MP1-MP6) was created based on the charge of the liposomes; for clarity, refer to Scheme 1. The composition and weight (in mg) of the lipids (DSPC, DDAB, and cholesterol), charged moieties, furazolidone, and NAC are provided in Table 1.

The lipid components DSPC and cholesterol, along with the charged moieties and Pluronic F-127 in the required formulations, were dissolved in chloroform. Furazolidone was separately dissolved in a minimum amount of acetonitrile (6 mg in 2 mL), which was then added to the lipid mixture. The solvent was evaporated in a rotary evaporator under reduced pressure for 15 min. At this stage, the system was purged with nitrogen, and the lipid layer was re-dissolved in a solvent for reverse-phase vesicles. A mixture of isopropyl ether and chloroform (2:1 *v*/*v*) was used as the solvent [49]. The aqueous phase was added to a system that already contained dissolved NAC at an organic/aqueous phase ratio of 3:1 [49,50].

The flask was sealed in nitrogen, and the mixture was sonicated for 20 min in a water bath until the mixture became a clear, one-phase dispersion. The mixture was incubated for 30 min to determine whether the system separated upon standing. The organic solvent was then evaporated under vacuum until the odor disappeared, followed by five freeze–thaw cycles, and the multilamellar vesicle (MLV) suspension was rapidly frozen to −196 °C using liquid nitrogen for 5 min, followed by thawing at 40 °C for 5 min. Rapid temperature changes induce the transformation of MLVs into large unilamellar vehicles (LUVs).

### 2.3. Mucopenetration Testing

For the mucopenetration test, six formulations were prepared using the same approach as REV; however, instead of encapsulating the drug, coumarin-6 was encapsulated in liposomes. Coumarin-6 was used as a marker fluorescent dye for this test to encapsulate the drug (a lipophilic dye that mimics the characteristics of furazolidone) to monitor the behavior of liposomal carriers using fluorimetry analysis. The use of coumarin-6 was not intended to track the diffusion of N-acetylcysteine (NAC), which is hydrophilic and is not the primary antibiotic against *H. pylori* in this study.

The free dye was separated from the entrapped dye by refrigerated centrifugation, followed by the separation of the green-colored layer from the white pellets. After centrifugation separation through a Sephadex G-50 column, the elution buffer was used.

A suspension of mucin type 1 (60 mg/mL) was prepared in distilled water. Mucin type 1 was chosen based on the literature and because it generally exhibits larger pore sizes than mucin type 3. A modified silicone tube method was used for mucus diffusion assay. Briefly, a silicon tube with a diameter of 8 mm and a length of 4 mm was used. The tube was inclined, and the mucin suspension was carefully added using a micropipette to avoid air bubbles. Three tubes were used for each formulation to estimate the diffusion after each hour. Once filled with mucin, a freshly prepared 20 µL liposomal suspension containing entrapped fluorescent dye was added, and the tube was incubated at 37 °C in a shaker incubator at 50 rpm for 1–3 h based on the time required.

At pre-determined time intervals (1, 2, and 3 h), the tube was removed and snap-frozen at −80 °C for two hours. After freezing, the tube was cut into 1 mm slices using a precision cutter and a Vernier caliper. Mucin and the liposomal suspension were removed from the cuts and diluted with 5 M NaOH (to dissolve mucin) and isopropyl alcohol (to disrupt the liposomal layer). The clear solution was analyzed by fluorimetry, and the intensity was used to calculate the percentage of diffusion in each millimeter slice by comparing it with the standard curve of coumarin-6.

### 2.4. Liposome/Particle Size Analysis

After five cycles of freezing and thawing, the expected particle size was less than 1 µm; therefore, a nanopore membrane of 500 nm and calibration particles of CPC 500 were used to cover the range of particle sizes from 300 to 800 nm. An Izon q-nano particle size analyzer (Izon Science Ltd., Christchurch, New Zealand) was used for size determination, as described by Alam et al. [11]. One calibration was performed for particle size analysis, which was then compared to the mucopenetrative liposome particles. The magnitude of the current blockage was measured and found to be directly proportional to the size of the particles [11]. The Izon q-Nanoparticle Size Analyzer uses tunable nanopores to determine the particle size as they pass through the pores. This method allows for the sizing of particles ranging from a few nanometers to micrometers, depending on the settings and type of nanopores used. In this study, a 500 nm nanopore was selected and calibrated using CPC 500 nm calibration particles. The 500 nm pore was ideal for selecting the range of the prepared liposomes suitable for experiments on mucin type 1.

### 2.5. Zeta Potential Measurement for Mucopenetration Liposomes

For the charge analysis, four different calibration runs were performed using the same nanopore and calibration particles (CPC) but at a pressure of 0 and 2 with an applied low voltage. The root-mean-square (RMS) noise was kept low throughout the run to generate reliable data. Control Suite V3.1 software was used for zeta potential determination using an Izon q-nano particle size analyzer (Izon Science Ltd., Christchurch, New Zealand).

### 2.6. Encapsulation Efficiency of Furazolidone and N-Acetyl Cysteine (NAC) within Mucopenetrative Liposomes

The encapsulation efficacy was assessed using a refrigerated centrifugation technique. The liposomal suspension was centrifuged at 4 °C for 10 min at 10,000 rpm. The supernatant was removed, and the pellets were washed with water to remove non-encapsulated NAC. However, to separate non-encapsulated furazolidone, the yellow compound was separated from the white pellet [11]. Once the liposomal suspension was free of unencapsulated drugs, the pellets were suspended in 5 mL of buffer at pH 7.4. The liposomal shell (lipid) was disrupted by adding isopropyl alcohol (IPA). After disrupting the lipid layer, the sample was injected into the HPLC system to determine the concentrations of furazolidone [11] and NAC. Once the concentration was determined, the percent encapsulation of both drugs was calculated based on the original amount of drug used.

### 2.7. HPLC Method for N-Acetyl Cysteine (NAC)

The NAC method was described by Ourique et al. [19]. However, this method does not require derivatization. Briefly, the sample containing NAC and lipids was dissolved in the mobile phase, which was 0.05 M KH_2_PO_4_ and acetonitrile (95:5 *v*/*v*), with the addition of 0.095% phosphoric acid (*v*/*v*). A total of 5% was used to stabilize the elution, and it was injected into a stationary phase column, which was C18 column (250 mm × 4.6 mm, 5 µm, 100 Å). The system used was an Agilent ChemStation LC-DAD equipped with a UV spectrophotometer (Santa Clara, CA, USA). A total of 100 µL of the sample was injected, and measurements were performed at 214 nm.

### 2.8. In Vitro Drug (Furazolidone and NAC) Release from Mucopenetrative Liposomes

The in vitro release of all formulations was determined using a modified dispersion method described by Alam et al. [11]. The release of the free drug was not performed because of its hydrophobicity, and the aim was to test the encapsulated furazolidone within the mucopenetrative liposomes. Analysis was performed for up to three hours at pre-determined intervals (15, 30, 45, 60, 90, 120, and 180 min). In this method, 1 mL of liposomal suspension was centrifuged by refrigerated centrifugation at 15,000 rpm for 10 min at 4 °C. The supernatant was discarded, and liposomal pellets were transferred to new vials without disturbing the yellow-colored furazolidone liposomes at the bottom. Liposomal pellets were washed thrice with water to remove unentrapped NAC and suspended in 5 mL of simulated gastric fluid (SGF) at the desired pH for the release experiment. After the specified time, 0.5 mL of the sample was withdrawn and replaced with an equal volume of fresh SGF, which was filtered through a 0.2 μm filter and then analyzed by HPLC analysis. The percentage of the drug released was calculated based on the encapsulated drug.

### 2.9. Transmission Electron Microscopy (TEM)

Liposome morphology was observed by TEM (Hitachi H7000 transmission electron microscope, Tokyo, Japan) using a negative staining technique with 1% (*w*/*v*) sodium silicotungstate solution to reveal the sphericity of the formed liposomes.

### 2.10. Reconstitution of Bacterial Culture

**Figure 1:**
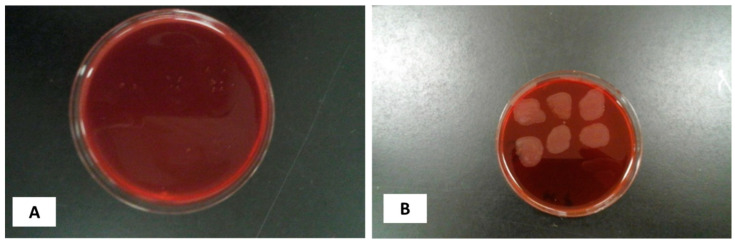
Spot plating technique for different dilutions from (**A**). 10^−1^ through 10^−3^ and (**B**). from 10^−1^ to 10^−6^.

To reconstitute, 0.5 mL of brain heart infusion (BHI) broth supplemented with 5% fetal calf serum was added to the ampule, followed by careful dissolution of the contents without producing any aerosol. The broth was maintained for 10 min to allow bacteria to rehydrate. They were then subcultured on blood and chocolate agar plates to obtain colonies on solid agar plates supplemented with DENT (vancomycin, trimethoprim, cefsoludin, and amphotericin B). For broth culture, fresh BHI broth containing 5% calf serum and DENT was inoculated with the reconstituted strain. All plates (Figure 1) and tubes were incubated in a 2.5 L Oxoid anaerobic jar with a CampyGen gas pack at 35 °C for 7 days.

### 2.11. Standard OD Controlled Growth and Inoculum Size

For growth quantification, different OD600 values were selected, and colonies were counted at each OD. An aliquot from the liquid culture prepared in the previous section was removed, and the OD600 was measured. The obtained value was adjusted to pre-determined OD600 values (0.05, 0.1, 0.3, and 0.5) using the following formula:

# mL = (target OD (600) × final culture volume)/(OD (600) of the starting culture)

# of mL = amount of liquid obtained from the original culture

The final culture volume was maintained at 2 mL.

The OD600 value of the starting culture was 0.53.

### 2.12. Time–Kill Curve of Augmented Therapy of Free and Liposomal-Bound Furazolidone with NAC

For the time–kill curve, the first minimum inhibitory concentration (MIC) of furazolidone and NAC was determined for the *H. pylori* strain (NCTC 12455) using the microbroth dilution method described in CLSI guidelines. The MIC values of both drugs were used to produce a time–kill curve for the augmented therapy.

A time–kill plot was constructed to determine the actual time needed by formulation MP1 (Table 1), free drug without formulation alone, and in combination with 1% minimum inhibitory concentration of NAC to kill the pre-determined concentration of the bacterial culture suspension. Briefly, the culture of *H. pylori* containing 5.5 × 10^5^ cfu/mL was incubated with different concentrations of free and liposomal-bound furazolidone. The minimum inhibitory concentration of NAC (1%) was added to all concentrations of furazolidone to study the modulatory effect of NAC on the MIC of furazolidone and determine the synergistic effect of both antibiotics on the killing time of *H. pylori*. Similar concentrations were used without 1% NAC as a control to confirm the effects of NAC. Cultures were incubated at 37 °C for 1.5, 2, 3, 6, or 8 h under microaerophilic conditions. The concentrations used for free furazolidone were 1/2, 4, and 8 × MIC, with a 1% minimum inhibitory concentration of NAC, which was 70 µg/mL. The CFU at each time point was counted after 24 h of incubation, and the log of CFU was plotted against time for each concentration used.

### 2.13. Statistic Analysis

All experiments were performed in triplicates. Data are presented as the mean ± standard deviation (SD). The significance of the differences was assessed using Excel version 9.1.0. Statistical significance was set at *p* < 0.05.

## 3. Results and Discussion

### 3.1. Characterization of Mucopenetrative Liposomal Formulations Regarding Their Encapsulation Efficiency and Liposome/Particle Morphology

The encapsulation efficiencies of all six formulations (Table 1) for both drugs are listed in Table 2, which shows the successful encapsulation of lipophilic and hydrophilic drugs into one vesicle using the reverse-phase evaporation method. This approach creates a high aqueous space-to-lipid ratio; therefore, REV can encapsulate a large amount of an aqueous-soluble drug. The maximum encapsulation efficiency was attained by the formulation MP6 (positively charged liposomes with no Pluronic F-127), which was 62% for NAC and 65% for furazolidone. Handa et al. [51] suggested that using the REV approach in general can increase the encapsulation efficiency by up to 85%.

**Figure 2:**
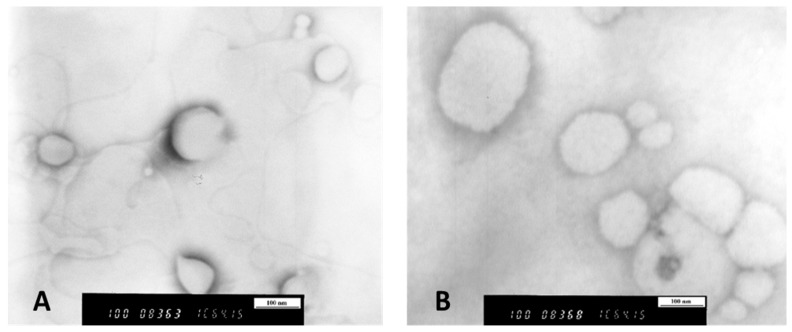
Transmission electron microscopic, TEM, images of mucopenetrative liposomes (**A**). MP3, cationic liposomes with Pluronic F-127 (**B**). MP1, neutral liposomes with Pluronic F-127. For formulations’ composition, refer to Table 1.

Figure 2 shows the TEM images of neutral and charged liposomes, which show the fusion of neutral liposomes (Figure 2B). However, the charged liposomes were more spaced out (Figure 2) because of the repulsion effect between vesicles, suggesting the colloidal stability of charged liposomes over neutral liposomes.

The encapsulation efficiency of the cationic liposomes was the highest, followed by that of the anionic liposomes (Table 2). However, neutral liposomes had the lowest encapsulation efficiency compared to charged liposomes. These data suggest that encapsulation depends on the electrostatic interactions between the charged lipid layers. This high encapsulation efficiency of NAC in charged liposomes could be due to the electrostatic repulsion between the lipid layers of multilayered liposomes, which in turn increases the aqueous cavity of liposomes, accommodating high amounts of water-soluble NAC [52]. NAC encapsulation was affected by the addition of Pluronic F-127 to the formulations. Both charged and neutral liposomes entrapped less NAC in aqueous space in the presence of Pluronic F-127. However, when Pluronic F-127 was not present in the formulations, all three formulations showed increased entrapment efficacy with maximum entrapment of 62%, suggesting that Pluronic F-127 may compete with the water-soluble NAC; hence, NAC encapsulation was less in the presence of the nonionic surfactant Pluronic F-127. However, the entrapment of furazolidone that accumulated in the lipid layers was not influenced by Pluronic F-127. The data suggested the successful encapsulation of sufficient quantities of the two drugs to achieve minimum inhibitory concentrations, as will be shown later in Section 3.4. Accordingly, we propose the use of liposomal combination therapy as an oral liposomal suspension dosage form. This will promote the safety and efficacy of furazolidone, which will act locally in the stomach, in a small dose to eradicate *Helicobacter pylori*.

### 3.2. Mucopenetration

**Figure 3:**
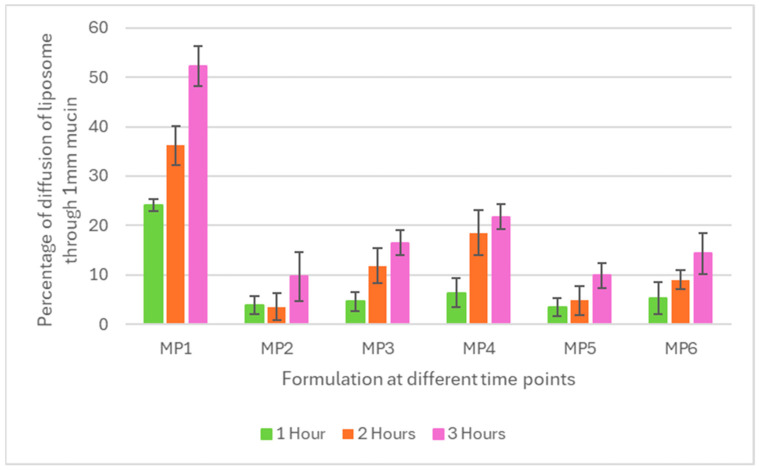
Diffusion of mucopenetrative liposomal particles from MP1 to MP6 through 1 mm thick sigma mucin type I in a silicon tube maintained at pH 6.0 at 37 °C at 1, 2, and 3 hours’ time (n = 3, mean ± SD shown). Abbreviations: MP1 (neutral with Pluronic F-127), MP4 (neutral without Pluronic F-127), MP2 (negative with Pluronic F-127), MP5 (negative without Pluronic F-127), MP3 (positive with Pluronic F-127), MP6 (positive without Pluronic F-127). For formulations’ composition, refer to Table 1.

Figure 3 shows the percentage of mucopenetration of six different formulations of reconstituted mucin type I at a concentration of 60 mg/mL at a depth of 1 mm. MP1 demonstrated the highest percentage of mucopenetration (52%), followed by MP4 (22%).

The liposomal formulation (MP1) achieved 24% in the first hour and 36% in the second hour, but the maximum penetration achieved was 52% after three hours. The experiment was conducted for three hours because the normal residence time in the stomach is approximately three hours. The liposomal formulation (MP4) exhibited a maximum of 25% after three hours. However, in the first and second hour, they were 6% and 18%, respectively.

According to the results of the current study, the formulation with no net charge penetrated up to 52% of the mucus. However, negatively and positively charged liposomes did not show promising results in terms of mucopenetration. Similarly, another study concluded that cationic liposomes adhere to negatively charged mucus threefold more than electrically neutral liposomes [53,54]. According to a previous study, electrically neutral particles diffuse more efficiently in cystic fibrosis sputum than anionic particles [55]. One study hypothesized that positively charged particles could bind to anionic glycosylated regions via polyvalent electrostatic interactions [56]. However, electrically neutral nanoparticles can easily diffuse into the mucus layer. It has been reported that both negatively and positively charged nanoparticles show limited diffusion compared to nanoparticles with a net neutral charge.

The triblock copolymer of poly (ethylene glycol)-poly (propylene oxide)-poly (ethylene glycol) (PEG-PPO-PEG; known as Pluronic F-127) has been shown to affect mucopenetration in different studies [55]. Pre-treatment of cervical mucus with Pluronic F-127 increased the penetration of nanoparticles through mucus [53,57,58]. Figure 3 shows the highest degree of mucopenetration achieved by MP1, which contains Pluronic F-127 and bears a neutral charge. This high percentage of mucopenetration could be due to the neutral charge or the presence of Pluronic F-127. Occasionally, electrically neutral nanoparticles are retarded in mucus owing to their hydrophobic nature; therefore, in addition to the electrically neutral charge, the hydrophilicity of liposomal formulations also contributes to the degree of mucus penetration. However, an increase in hydrophilicity can enhance mucus penetration [58,59].

Therefore, the addition of Pluronic F-127 in the current study imparted hydrophilicity to the liposomal formulation, which in turn increased mucus penetration. Second, the effect of Pluronic F-127 on mucopenetration was only obvious in MP1, whereas its effect was not clear in MP2 and MP3, which had the same concentration of Pluronic F-127.

The apparent reason for this seems to be the presence of charged moieties that mask the mucopenetrative effect of Pluronic F-127. The effect of Pluronic F-127 on mucus penetration has been explained in a study that showed that the treatment of nanoparticles with Pluronic F-127 can improve the diffusion rate [57,60].

NAC is a mucolytic agent that has been shown to improve mucus penetration by nanoscale particles and liposomes [53,61]. The results indicated a substantial increase in mucus penetration of liposomal formulations containing NAC with no net surface charge (MP1). These findings are in agreement with another study conducted by Ferrari et al. [35] who reported that partial improvement of non-viral gene vectors could improve the use of NAC in a sheep tracheal model [35]. The mucolytic properties of NAC can be attributed to multiple mechanisms. This can reduce the bulk rheology of mucus [53,62]. It replaces the disulfide bond with a thiol group that connects mucin proteins, which in turn reduces the viscosity of mucin [62,63]. The results shown in Figure 3 indicate that the effect of charge was more influential than that of NAC. All six formulations contained equal amounts of NAC with different surface charges. Only MP1 and MP4, which had no net charge, showed good diffusion through mucin. However, there was no considerable diffusion through the mucus in the other formulations. Similarly, the use of NAC alone in formulation MP4 resulted in less penetration than MP1, which contained both NAC and Pluronic F-127. This finding indicated that both Pluronic F-127 and NAC in a nanoform combination had a synergistic effect on mucus penetration.

The size of liposomes is an important factor that must be considered to allow the particles to diffuse smoothly through mucus. Mucus has a mesh size of gastric mucin ranging from 200 to 650 nm [53,64]. The particle size of liposomes in this study ranged from 350 to 800 nm, as listed in Table 2, which potentially enables them to diffuse through the mucus. The mucopenetration experiment was carried out at pH 6.0 because the desired pH in this study was selected to be alkaline by considering the presence of *H. pylori*. The presence of *H. pylori* and the use of a proton pump inhibitor increased the stomach pH to a range of 6–7 [65]. This alkaline pH enhances penetration, as described in a study in which an acidic pH below 4 increased mucus viscosity [66,67]. Another study explained the relationship between pH and bulk viscosity of mucus, stating that a decrease in pH from 6 to 4 increases the viscosity and bulk moduli by up to 1000 folds in magnitude [65]. Therefore, maintaining an alkaline pH using a proton pump inhibitor increases the mesh size and facilitates the diffusion of neutral particles through mucus. Treatment of mucus with NAC increased the mesh size of native mucin from 300 nm to 1300 nm [68,69] therefore, the prepared mucopenetrative liposomes were able to diffuse through mucus.

The combined effect of charge and Pluronic F-127 was statistically analyzed, and the results proved that the effect of charge, as well as Pluronic F-127, was significant (*p* < 0.05) at all time points. According to the post hoc Tukey test, the effect of charge was less influential in mucopenetration with Pluronic F-127. The neutral formulation, in the absence of Pluronic F-127, showed high diffusion (21% after 3 h) compared to positively (14% after 3 h) and negatively charged (9% after 3 h) liposomes. However, negatively charged liposomes showed a lower degree of penetration than positively charged liposomes. Similarly, in the case of Pluronic F-127 added to the formulations, the effect of charged particles on mucopenetration was significantly reduced (*p* < 0.05) compared to that of neutral particles. Neutral particles demonstrated 52% diffusion after three hours, whereas the positive and negative formulations showed only 16% and 9% diffusion, respectively. Therefore, in relation to each other, the presence or absence of Pluronic F-127 does not affect positively and negatively charged formulations after three hours.

### 3.3. In Vitro Drug Release of Mucopenetrative Liposomes

#### 3.3.1. In Vitro Drug Release of Furazolidone

**Figure 4:**
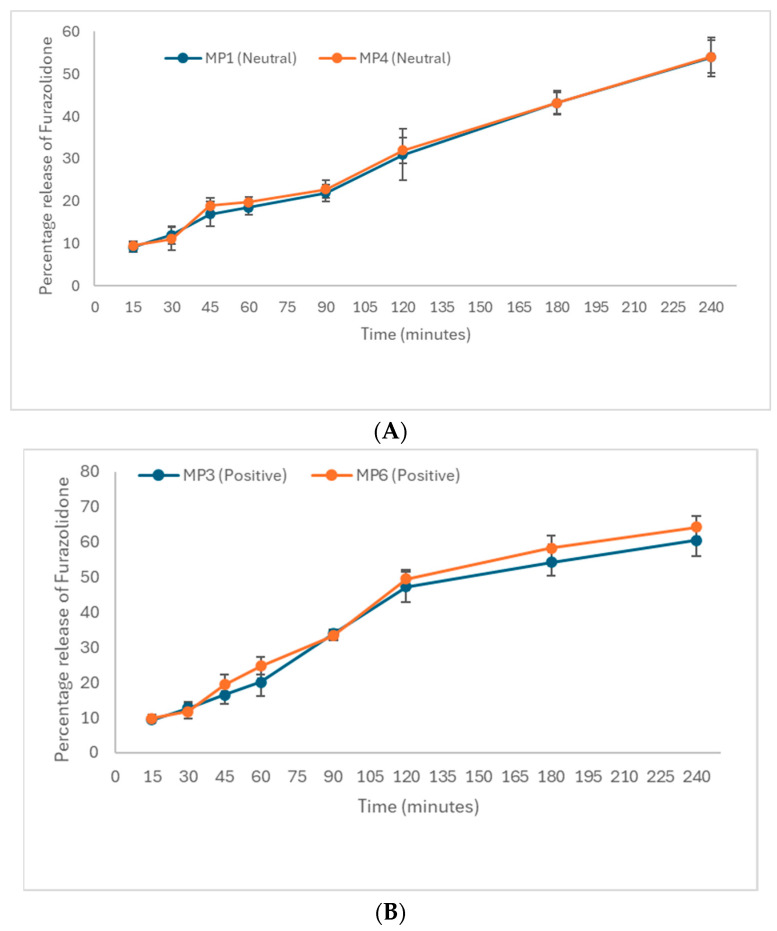

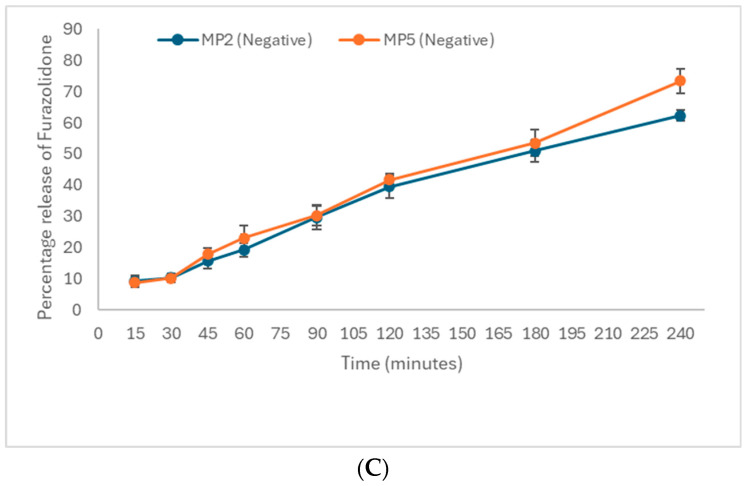
(**A**). In vitro drug release of furazolidone from the mucopenetrative formulation MP1 and MP4 up to a 4 h time at pH 6.0 (n = 3, mean ± SD shown). Abbreviations: MP1 (neutral with Pluronic F-127), MP4 (neutral without Pluronic F-127). (**B**). In vitro drug release of furazolidone from the mucopenetrative formulation MP3 and MP6 up to a 4 h time at pH 6.0 (n = 3, mean ± SD shown). Abbreviations: MP3 (positive with Pluronic F-127), MP6 (positive without Pluronic F-127). (**C**). In vitro drug release of furazolidone from the mucopenetrative formulation MP2 and MP5 for up to a 4 h time at pH 6.0 (n = 3, mean ± SD shown). Abbreviations: MP2 (negative with Pluronic F-127), MP5 (negative without Pluronic F-127).

The release of furazolidone and NAC from the formulations was analyzed at three different time points to determine the effect of charge and Pluronic F-127 on the release behavior of liposomes. After 30 min of the in vitro drug release test, there was no significant effect of Pluronic F-127 on release (*p* = 0.138). As shown in Figure 4A–C, the release rates of the first three formulations with Pluronic F-127 were almost similar to their counterparts without Pluronic F-127. However, the effect of charge was significant. Both formulations bearing a positive surface charge, MP6 and MP3, showed a maximum release of 12% after 30 min. However, the neutral formulations MP1 and MP4 released 12% and 11%, respectively, which was slightly less than the positive formulations. The negatively charged formulations MP2 and MP5 released the lowest amount of drug within 30 min. After 60 min of drug release, the effect of Pluronic F-127 was significant. At 60 min, the positively charged formulations MP6 and MP3 released 25% and 20% furazolidone, respectively).

However, 23% of the drug was released by MP5, and 19% of the drug was released from MP2, which showed less release compared to positively charged liposomes MP6. In the case of neutral liposomes (MP1, MP4), drug release of the drug remained unaffected by the presence of Pluronic F-127, as shown in Figure 4, where both MP1 and MP4 released almost 18–19% of the drug. Similarly, at 240 min, the formulations with Pluronic F-127 (MP2 and MP3) released less drug in the presence of charged moieties and demonstrated unaffected drug release in neutral liposomes.

#### 3.3.2. In Vitro Drug Release of N-Acetyl Cysteine, NAC

**Figure 5:**
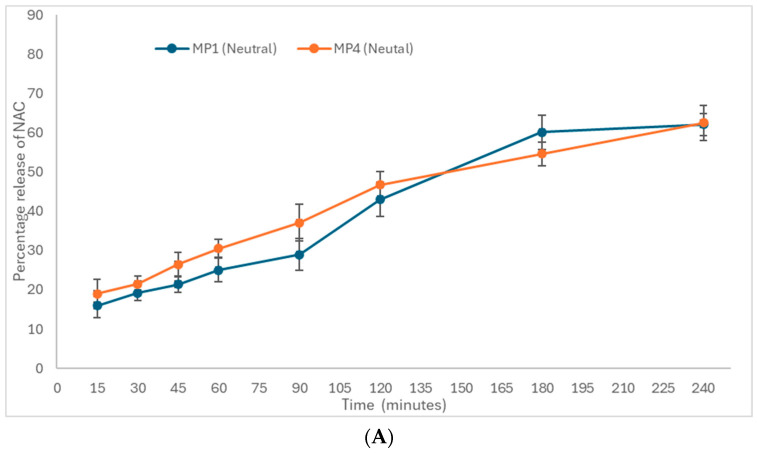

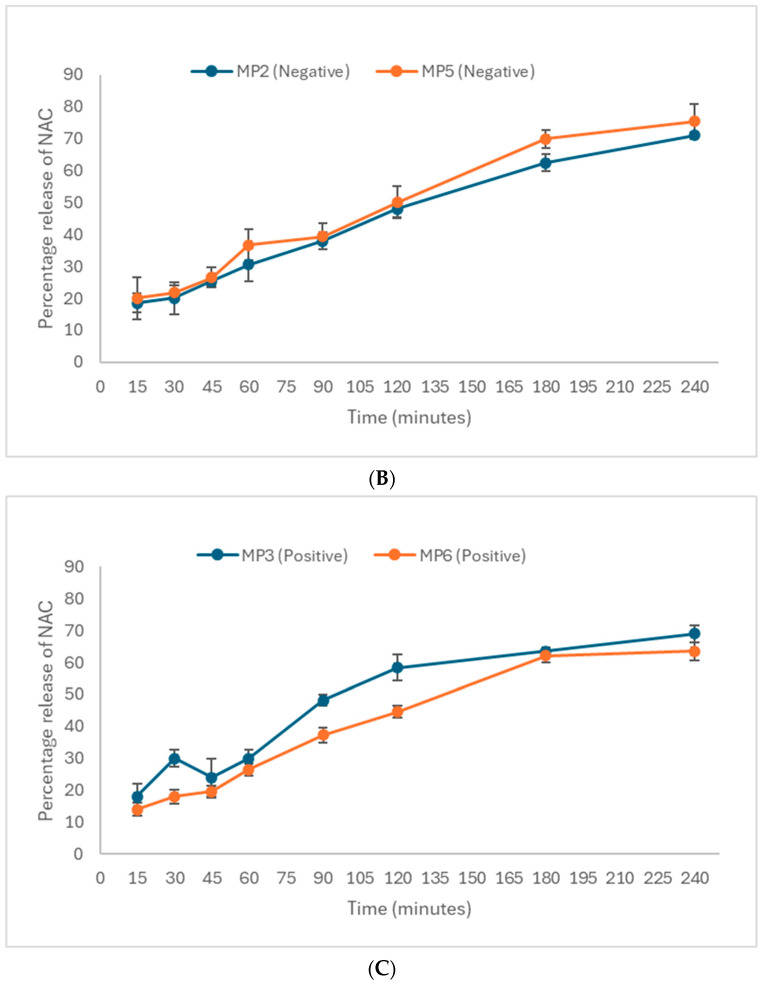
(**A**). In vitro drug release of NAC from the mucopenetrative formulation from MP1 and MP4 for up to 4 h time at pH 6.0 (n = 3, mean ± SD shown). Abbreviations: MP1 (neutral with Pluronic F-127), MP4 (neutral without Pluronic F-127). (**B**). In vitro drug release of NAC from the mucopenetrative formulation from MP2 and MP5 for up to 4 h time at pH 6.0 (n = 3, mean ± SD shown). Abbreviations: MP2 (negative with Pluronic F-127), MP5 (negative without Pluronic F-127). (**C**). In vitro drug release of NAC from the mucopenetrative formulation from MP3 and MP6 for up to 4 h time at pH 6.0 (n = 3, mean ± SD shown). Abbreviations: MP3 (positive with Pluronic F-127), MP6 (positive without Pluronic F-127).

In the case of NAC, which is a hydrophilic drug that tends to remain in the aqueous cavity, the effect of Pluronic F-127 on the release profile was inconsistent. On the other hand, the effect of Pluronic F-127 on furazolidone, which is a hydrophobic drug that stays in the lipid layers, is significant (*p* < 0.05) in terms of drug release from charged liposomes compared to neutral liposomes. The effect of charge on the release of NAC demonstrated that negatively charged liposomes provided maximum release at all selected time points when compared with positively charged and neutral liposomes, as shown in Figure 5A–C.

In the current study, the release rates of the drug (furazolidone) from positively charged liposomes were higher in the first two hours compared to the negative and neutral liposomal formulations. These findings are supported by a study that reported a high release from positively charged liposomes in the first two hours [70].

The results of the in vitro drug release for both drugs (furazolidone and NAC) were consistent with a study that stated that negatively charged liposomes released the maximum amount of drug compared to positive and neutral liposomes [71]. This fact is also mentioned in another study, which stated that imparting a negative charge to liposomes enhances the release rate of neutral particles at alkaline pH, similar to the current study conducted at pH 6.0 [70,72].

The release rate of NAC from neutral liposomes was lower at the final time point compared to that from negative and positively charged liposomes, which agrees with the results showing that the release of doxorubicin was less than that from anionic and cationic liposomes [73]. However, the neutral liposomes initially showed a high release in the first two hours. A possible explanation could be the short duration of sampling at the start of the experiment.

### 3.4. Time–Kill Curve of Augmented Therapy of Free and Liposomal-Bound Furazolidone with NAC

**Figure 6:**
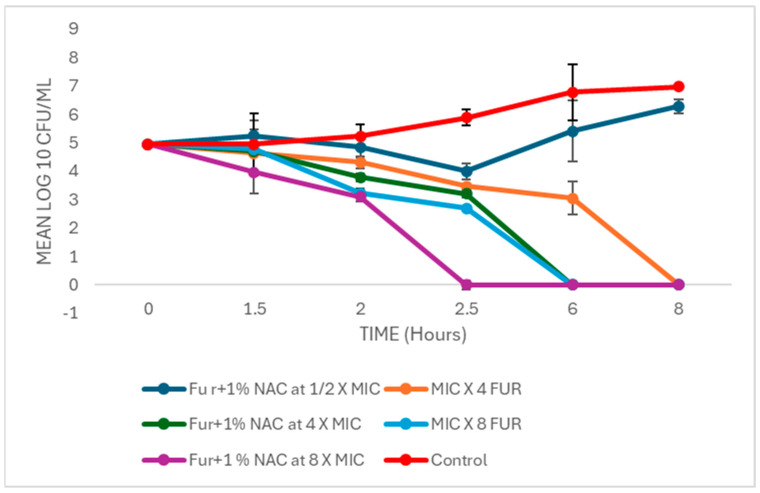
A time–kill curve experiment of furazolidone augmented with NAC at a concentration of 1% of its minimum inhibitory concentration (MIC) against *H. pylori* inoculated on a blood agar plate and incubated at 37 °C for 24 h in an anaerobic jar with CampyGen gas packs before counting the colonies. The mean value of the log number of cfu per milliliter was plotted against time (n = 6, mean ± SD).

Figure 6 shows the time–kill curve of the liposomal formulation and free furazolidone in combination with 1% MIC of NAC, which achieved complete killing in 2.5 h.

The time–kill curve in Figure 5 shows that half of the MIC (2 µg/mL) of furazolidone in combination with 1% MIC of NAC demonstrated an initial drop of 1 cfu/mL in the colony count to 3 h, but regrowth was observed up to eight hours of incubation. A similar phase of regrowth was observed in a study by Rukholm et al. [74], in which the antibiotic was unable to maintain antimicrobial performance at its MIC after 6 h. This indicated that the combined effect of the sub-inhibitory concentration of furazolidone with NAC was not sufficient to induce significant cell death. Higher concentrations caused a significant drop in the log; that is, by increasing the concentration (4 × MIC) of furazolidone, complete killing was observed after eight hours of incubation. This indicates that the inhibition of *H. pylori* is concentration-dependent. However, the same concentration of furazolidone, when used in combination with 1% NAC, was completely efficient to kill *H. pylori* in 6 h rather than 8 h. These results could be explained by the fact that NAC at lower concentrations reduced the MIC of furazolidone (<4 µg/mL). Therefore, furazolidone used at the same concentration in the presence of 1% NAC reduced the time required to kill the bacterial cells (*H. pylori*). The modulatory effect of NAC, when used in combination, reduced the MIC of carbenicillin from 16 to 1 µg/mL, as reported in another study by Zhao and Liu [75]. Similarly, in 2010, Goswami et al. [76] reported that NAC could be used as a modulator of different antibiotics. The results of this study are in agreement with the hypothesis that NAC augments the activity of antibiotics to reduce *H. pylori* cells [77,78]. However, the kinetics of the killing curve showed that the use of an increased concentration of furazolidone alone by MIC × 8) effectively killed the bacterial cells as quickly as the killing observed using furazolidone 4 × MIC in combination with 1% NAC. These results are supported by another study, in which the use of an increased concentration of amoxicillin or a combination of amoxicillin and glycine as a modulator increased the rate of *H. pylori* [79,80]. The mechanism of modulation is still not clear, but it could be due to the thiol group present in cysteine, which may react with cellular proteins [75].

However, when the formula was used in a liposomal formulation, 8X MIC of furazolidone and 1% MIC of NAC achieved complete killing of bacterial cells within 2.5 h, which is the resistance time of the unmodified dosage form in the stomach under normal physiological conditions [81].

## 4. Conclusions and Future Perspective

Liposomes capable of encapsulating two drugs (furazolidone and NAC) and penetrating through mucus were successfully formulated, and their augmented effect on *H. pylori* residing in stomach mucus was demonstrated. The formulations were categorized into three broad groups based on their charges, which were achieved by incorporating the charged moieties. These groups were further subdivided based on the presence or absence of Pluronic F-127. Charged liposomes exhibited higher encapsulation efficiency than neutral liposomes for both drugs. The encapsulation efficiency of neutral liposomes was unaffected by the addition of Pluronic F-127.

The formulation MP1 (neutral with Pluronic F-127) was considered the most effective in terms of mucopenetration, showing the highest percentage of penetration. However, for drug release from liposomes, MP5 exhibited the highest percentage of drug release.

From the killing curves, it is evident that the inhibition of *H. pylori* is concentration-dependent. Formulations containing furazolidone took longer to completely inhibit bacterial growth compared to formulations containing 1% NAC. Increasing the concentration of furazolidone in formulations with 1% NAC reduced the killing time accordingly.

Further investigation into the storage stability of mucopenetrative liposomes and their release profiles under more clinically relevant conditions, such as varying pH and ionic strength, is necessary to better simulate in vivo environments. Additionally, conducting in vivo studies to evaluate the effectiveness and safety of these liposomal formulations in a physiological context is crucial to translating these findings into clinical applications. Future studies should also explore the potential of combining liposomal delivery systems with other adjunctive therapies to enhance the overall treatment of *H. pylori* infections, particularly in cases with high antibiotic resistance.

## Figures and Tables

**Table 1:** Composition of mucopenetrative liposomal preparation. All components were weighed in milligrams.

Liposomes	Coding	Lipid/DSPC	Cholesterol	Total Lipid	DDAB	DCP	Furazolidone	NAC	Pluronic F-127
Neutral	MP1	58.5	6.5	65	-	-	6	14	5
Neutral	MP4	58.5	6.5	65	-	-	6	14	-
Negative	MP2	57.5	6.5	65	-	1	6	14	5
Negative	MP5	57.5	6.5	65	-	1	6	14	-
Positive	MP3	57.5	6.5	65	1	-	6	14	5
Positive	MP6	57.5	6.5	65	1	-	6	14	-

Dihexadecyl phosphate (DCP), didodecyldimethylammonium bromide 98% (DDAB), N-Acetyl cysteine (NAC).

**Table 2:** Encapsulation efficiency, zeta potential, and liposomal size of mucopenetrative liposomal formulations.

Formulation	Encapsulation Efficiency (%)		Zeta Potential (mV)	Particle Size (nm)
	Furazolidone	NAC		
MP1	58.9 ± 3.4	51.41 ± 1.2	+3.5	490
MP2	62.2 ± 3.2	52.35 ± 0.9	−19.1	520
MP3	67.9 ± 3.8	54.25 ± 1.5	+10.3	600
MP4	56.0 ± 4.2	50.53 ± 2.1	−1.4	530
MP5	63.9 ± 6.5	59.52 ± 1.2	−36.1	570
MP6	65.6 ± 7.5	62.45 ± 2.3	+7.2	740

## Data Availability

Data are contained within the article.
